# How much disease risk is due to old age and established risk factors?

**DOI:** 10.1093/pnasnexus/pgad279

**Published:** 2023-09-12

**Authors:** A J Webster

**Affiliations:** Nuffield Department of Population Health, Big Data Institute, University of Oxford, Old Road Campus, Oxford OX3 7LF, UK

**Keywords:** multimorbidity, age, exposures, multistage, Poisson Binomial

## Abstract

Improved healthcare is leading to older populations and increasing numbers of individuals experiencing multiple diseases, possibly concurrently (multimorbidity). This article asks whether the observed number of new diseases is more than expected based on age and established risk factors alone, assuming that disease risk is unchanged by prior or pre-existing disease. This is accomplished by designing a new epidemiological approach, where the expected number of disease types are estimated for individuals without prior disease, by combining individual risk predictions with a “Poisson-Binomial” model to estimate the expected number of new diseases and its confidence interval. For 123 diseases in men and 99 diseases in women, the expected number of new diseases based on age and established risk factors was approximately 2/3 of that observed, with the observed number of new diseases approximately 1.5 times that predicted. The differences could not be explained by natural statistical variation, and provide a rigorous statistical demonstration of lower disease risk for individuals without any previous disease. The multiple of 1.5 was sufficiently consistent across different diseases to prevent its use for classification of disease types, but there were differences for subgroups such as smokers with high body mass index, and for some classes of disease (as defined by the International Classification of Diseases, version 10). The results suggest that empirical modeling might allow reliable predictions of future hospital admissions, and confirm the value of conventional epidemiological approaches that study disease risk in healthy individuals. The implications and future possibilities of this new approach are discussed.

**Significance Statement**

It is widely believed that disease risk can be increased by the presence of one or more (different) existing diseases. As improved healthcare leads to older populations, more people will experience multiple and sometimes coexisting diseases (multimorbidity), amplifying the need to understand and predict future healthcare demand. This article provides the first statistically rigorous attempt to quantify how previous or pre-existing diseases increase the risk of a broad range of over 100 common diseases in the UK Biobank cohort. The methods can be applied more widely, and it is hoped that this will be the first of increasingly comprehensive population-wide studies.

Multimorbidity is increasingly common in developed economies such as the United Kingdom. It is widely believed to involve clusters of diseases and that disease risks are modified by underlying conditions (1–4). Despite considerable work to characterize multimorbidity in terms of clusters of diseases (e.g. see the recent review (4)), there have been comparatively few recent studies to determine the influence of preexisting conditions on the age-dependent incidence rates of disease (2, 5–11). Most statistical studies (4) consider complex sequences of disease in individuals, possibly confounded by several external factors and a range of medications. Unfortunately it is extremely challenging to predict the influence of preexisting diseases on future disease risk using this approach, and conceivably, it may not be possible.

Here we take a different approach, and firstly consider the much easier problem of predicting how much disease incidence we would expect to see based on age and established risk factors, in disease-free individuals. This is the situation considered in most epidemiological studies, that estimate the influence of risk factors on disease risk in healthy individuals to avoid confounding by prior or pre-existing diseases such as cancer, that would be expected to bias and invalidate estimates. By following an initially healthy cohort, and comparing the expected incidence of new diseases in disease-free individuals with the actual disease cases experienced by the cohort, we can explore how prior or pre-existing disease is modifying disease incidence from what we would expect in disease-free, otherwise healthy individuals.

The approach involves two steps, firstly to predict disease incidence in disease-free individuals in terms of their age and established risk factors, for which there are already several established methods. The second step uses these predictions to estimate how many cases of new disease types we would expect to see. This is analogous to predicting the number of disease counts using a Poisson distribution, but here the risks are different for each individual and for each disease. The mathematical solution to this problem is outlined in the Materials and methods section and involves a little-known statistical distribution called the “Poisson-Binomial” distribution (12, 13). This allows the expected number of disease cases to be predicted with a confidence interval, and to rigorously test whether the observed number of new diseases are consistent with the null hypothesis that disease risk is equivalent to that of disease-free individuals.

A recent study (14) used a Weibull distribution to explore whether the age-related incidence of diseases in UK Biobank (15) are consistent with multistage disease processes (14, 16–18). This model, dataset, and disease definitions are used here because: (1) they allow a high-quality study using established methods with results that have recently been published in several peer-reviewed articles (14, 18, 19), and most importantly, (2) because a Weibull model allows predictions to be extrapolated beyond the duration of the study period. This would not be possible for a nonparametric model such as a Cox proportional hazards model, and can allow future studies to use competing-risk models to explore future healthcare demands. An outline of the analysis is in Fig. 1.

**Fig. 1.**
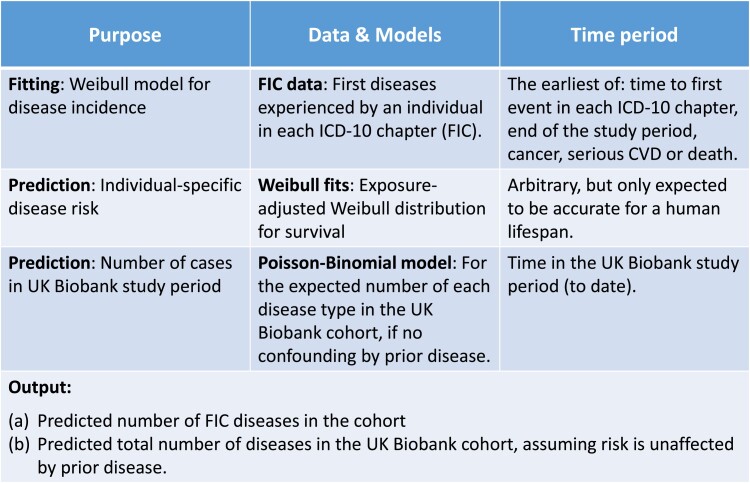
The table summarizes the data and analysis methods used (top to bottom).

The method requires sufficient data to allow meaningful statistical comparisons between the observed and expected number of cases, and a dataset with information on important risk factors such as smoking and body mass index (BMI). The UK Biobank includes detailed phenotypical information on a cohort of approximately 500,000 individuals, with information on established risk factors and hospital disease diagnoses through linked hospital records. It was used here in preference to other large datasets because it is well understood (15), and was used in our previous studies where the study design (20) and efficacy of the Weibull model were demonstrated (14). The data allows a longitudinal prospective study design, with a time-to-event analysis that accounts for well-known risk and confounding factors to estimate the age-related disease risk in disease-free individuals. A strength of the approach is that it does not rely on the sequence of disease presentation, which cannot always be reliably determined from hospital records. The most common 800 diseases in UK Biobank data were considered, of which 450 were consistent with the Weibull model, and there were sufficient cases for 172 (or 156) to be modeled with adjustment for 7 (or 9) established risk factors in men (or women) (14).

An outline of the article is as follows. The statistical method is described next, with the results presented and discussed in the following two sections. We will find that the observed number of new disease cases are well above that expected based on estimates for disease-free individuals, with approximately two thirds of cases explained by age and the established risk factors considered here. The size of increases will be found to be surprisingly similar for men and women, and across different disease types, although we will find differences for some classes of disease types and for subgroups of individuals such as smokers with high BMI. The results emphasize the substantial extra healthcare burden due to known risk factors such as smoking and BMI, contributing to an increasingly persuasive case for strong policy interventions to reduce healthcare demand. They also offer the tantalizing suggestion that simple empirical models may allow reliable prediction of future healthcare need. The results and their implications are explored in more detail in the Discussion section, and the key findings are summarized in the Conclusions section.

## Methods

### Statistical background

We test the null hypothesis that the incidence rate of each disease type is independent of the presence of different previous, or coexisting diseases. Using incidence rates of each disease type, that are estimated for a scenario that approximates no prior disease, we calculate the probability of observing $N_{j}$ cases of disease type *j* in the UK Biobank cohort when there is no prior disease. Let $S_{ij}(t)$ be the probability of person *i* surviving disease *j* until age *t*, then $p_{ij}=S_{ij}(t_{i}^{start})-S_{ij}(t_{i}^{end})$ is the probability of person *i* first experiencing disease *j* between the ages $t_{i}^{start}$ and $t_{i}^{end}$ during which they were in the study (that equals the integral of the probability density function for the age at first incidence of disease *j* between ages $t_{i}^{start}$ and $t_{i}^{end}$). Let $X_{ij}=1$ if individual *i* has disease *j*, and zero otherwise. Then if disease risks are independent, the probability of i=1 to i=n individuals observing the set of diseases $\left\{X_{ij}\right\}$ is


(1) $$P(X_{1j}=x_{1j},\ldots ,X_{nj}=x_{nj})=\Pi_{i}p_{ij}^{x_{ij}}(1-p_{ij}{)}^{1-x_{ij}}$$

The probability of observing $N_{j}=\sum_{i}X_{ij}$, where $N_{\;j}$ is the number of individuals who experience disease of type *j*, is given by the “Poisson-Binomial” distribution,


(2) $$\begin{array}{rl} & P\left(\sum_{i}X_{ij}=N_{j}\right)\\ & \;=\sum_{\left\{x_{ij}=0,1\right\}}\delta \left(N_{j}-\sum_{i}x_{ij}\right)\Pi_{i}p_{ij}^{x_{ij}}(1-p_{ij}{)}^{1-x_{ij}} \end{array}$$

where δ(s) is the Dirac delta function that equals 1 when s=0 but is zero otherwise, and the sum is over all values $x_{ij}=0$ and 1, for i=1…n. The mean and variance of the distribution are


(3) $$E[N_{j}\;|\;\{p_{ij}\}]=\sum_{i}p_{ij}$$

and


(4) $$Var[N_{j}\;|\;\{p_{ij}\}]=\sum_{i}p_{ij}(1-p_{ij})$$

Simple derivations of these results are given in the Materials and methods section. For $p_{ij}\ll 1$, as is the case for most of the diseases in most individuals considered here (14, 19, 21), then the distribution will approximate a Poisson distribution, which provides another reason why the Poisson distribution is common—it approximates the distribution for the number of events arising from rare independent processes. If in addition $1\ll \sum_{i}p_{ij}$, then the Poisson distribution can be approximated by a normal distribution with mean and variance of $\sum_{i}p_{ij}$.

For each disease *j* and individual *i*, the multivariate δ-method (22) allows variances $\sigma_{ij}$ to be estimated for maximum likelihood estimates (MLEs) for the probability ${\hat{\;p}}_{ij}$ for disease *j* during the time observed, with ${\hat{\;p}}_{ij}∼N(p_{ij},\sigma_{ij}^{2})$. The law of total variance states that,


(5) $$Var[N_{j}]=E[Var(N_{j}\;|\;\{p_{ij}\})]+Var[E(N_{j}\;|\;\{p_{ij}\})]$$

that can be evaluated using Eqs. 3 and 4. Surprisingly perhaps, the variances $\sigma_{ij}^{2}$ that arise from integrals involving $p_{ij}^{2}$ cancel, and do not appear in the result, that is,


(6) $$Var[N_{j}]=\sum_{i}p_{ij}(1-p_{ij})$$

and can be estimated by “plugging in” the MLEs $\left\{{\hat{\;p}}_{ij}\right\}$ for $\left\{p_{ij}\right\}$. In practice the estimated variances $\left\{\sigma_{ij}^{2}\right\}$ were important for “quality control”, allowing the identification of poor-quality estimates that are too imprecise, or unlikely to satisfy the assumptions needed for application of the δ-method. Specifically, diseases were excluded if estimates for parameters x=k or x=L had s.e.(x)/x>0.5, or where the δ-method failed to give a numeric estimate, and it was confirmed that the remaining estimates had $\sum_{i}\sigma_{ij}^{2}/N_{j}<0.05$. The negative correlation between parameters *k* and *L* led to smaller variances for estimates than might have been expected based on estimates for the variances of *k* and *L* individually.

### Data analysis

Diseases included in the study were defined as a collection of one or more 3- and 4-digit disease codes from the International Classification of Diseases Version 10 (ICD-10) (23), that were selected by three epidemiologists with individual backgrounds in pathology, general practice, and statistics, based on a set of predetermined criteria as detailed previously (20). Diagnoses used primary cause of admission in hospital episode statistics, that ensured that the diseases had passed a threshold of severity prior to diagnosis. The study period for an individual was between joining the study and 2020 January 31, after which admission rates will be influenced by the Covid pandemic. To minimize the potential influence of prior diseases or medications, individuals were excluded if they had a prior report (either self-reported or in hospital records) of cancer other than non-melanoma skin cancer before the study started, or of serious cardiovascular disease (CVD). Provided there are no unmeasured confounders that differ substantially between the excluded and nonexcluded populations, then the exclusions are accounted for in the statistical analyses and should not bias the results or limit their generalisability. This common epidemiological procedure is necessary to reduce risk of bias due to potential consequences of cancer or serious CVD and their treatment. Sensitivity analyses were used to explore the potential influence of this assumption.

The study considered all primary diagnoses within the study period (“all cases”), and similar to previous work (14, 19, 20), the first primary diagnosis that an individual receives in each ICD-10 chapter (“FIC”). By considering only the first diagnosis in each ICD-10 chapter, the intention was to minimize confounding by prior disease, while maximizing the number of cases that are included in the study. Importantly, because the study considers the number of different diseases experienced by an individual, counts do not include repeat admissions for the same disease in the same individual.

A previous study of UK Biobank data identified 450 diseases whose FIC incidence rates could be modeled by a Weibull distribution (14),


(7) $$S(t)=\exp\;\left(-e^{x^{T}\beta }{\left(\frac{t}{L}\right)}^{k}\right)$$

where *t* is age, *k* and *L* are parameters, and *x*, β are vectors of covariates and parameters. Individuals were excluded if they had a report of the disease before the study’s start. MLEs and their covariances were calculated by left-truncating at the age when participants joined the study, taking age of event as the age of disease onset, and right-censoring if there is cancer, death or the study ends before disease onset. These procedures estimate the probability distribution for age at first diagnosis of the studied disease. Adjustment was for the established risk factors of smoking (never, previous, current), diabetes (yes, no), alcohol (rarely—less than 3 times per month, sometimes—more than 3 times per month but less than 3 times per week, regularly—more than 3 times per week), deprivation tertile (min, mid, max), education (degree level, post-16, to age 16), and sex-specific tertiles of height and BMI (min, mid, max). Baseline was never smoked, no diabetes, sometimes drink, min deprivation, degree-level education, middle BMI, minimum height and deprivation tertiles, and women with no use of HRT (Hormone Replacement Therapy), and no children.

**Fig. 2.**
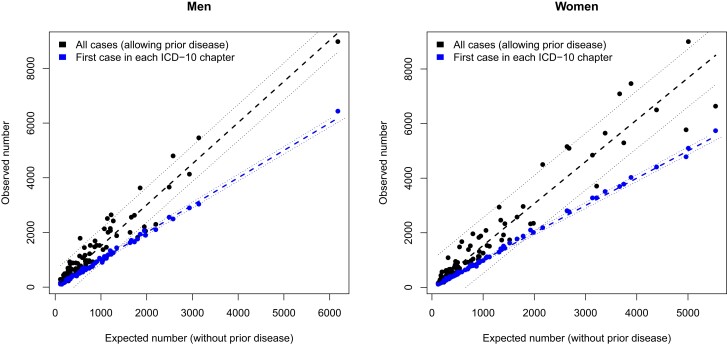
For each disease, the number of individual incidences are predicted in the absence of prior disease (Expected number), and plotted against the total observed number of cases (black) and observed number of cases where the disease is the first an individual experiences in each ICD-10 chapter (blue/grey). 95% confidence intervals are estimated using Eq. 6 as $\pm 1.96\sqrt{\text{Var}}$ (blue), and for predictive confidence intervals of the linear fit (black).

For each disease whose age-dependent incidence could be modeled sufficiently well, the observed number of cases in the UK Biobank cohort was plotted versus the number of predicted cases assuming no confounding by prior disease (Fig. 2). The results for men and women were plotted separately, and excluded diseases whose predicted case numbers differed by more than 4 standard deviations (sd) from the observed numbers of first diseases in each ICD-10 chapter. The (lenient) threshold of 4 sd was intended to include as many diseases as possible, while limiting the risk of outliers from substantially influencing subsequent results. For example when $p_{ij}\ll 1$ and $1\ll \sum_{i}p_{ij}$, then $p_{ij}\ll 1$ allows us to approximate the Poisson-Binomial distribution with a Poisson distribution, and for $1\ll \sum_{i}p_{ij}$ the Poisson distribution approximates a normal distribution whose mean and variance equal $\sum_{i}p_{ij}$. For those diseases 99.9% of them would be expected to have the observed number of first admissions in each ICD-10 chapter within 4 sd of the predictions.

Confidence intervals are reported as ±1.96σ, where σ is the estimated standard deviation of the MLE. For an MLE ${\hat{\beta }}_{i}$, ${\hat{\beta }}_{i}∼N(\beta_{i},\sigma_{i}^{2})$, and tests for equality of MLEs use $\left({\hat{\beta }}_{i}-{\hat{\beta }}_{j})∼N(0,\sigma_{i}^{2}+\sigma_{j}^{2}\right)$, so statistically significant differences at the 0.05 level will have $|{\hat{\beta }}_{i}-{\hat{\beta }}_{j}|>1.96\sqrt{\sigma_{i}^{2}+\sigma_{j}^{2}}$.

## Results

Figure 2 plots the number of each disease type observed in the UK Biobank cohort versus the predicted number of cases if there is no confounding by prior disease, with poorly modeled data removed (as described above). A straight-line fit through the origin estimated the number of observed diseases in men to be 1.50 [1.45,1.55] times the expected number of diseases without prior disease, and 1.53 [1.47,1.59] in women (Tables 1 and 2). There is no statistically significant difference between the fits for men and women. The *R*-squared value for both fits was 0.96. Data for individual diseases are given in online supplementary Tables S1 and S2.

**Table 1.** Men: Increased incidence associated with prior disease.

Group	Coef	C.I.	*R*-squared
Everyone	1.50	[1.45,1.56]	0.96
Nonsmoker, mid-BMI	1.52	[1.46,1.58]	0.95
Smoker, mid-BMI	1.56	[1.48,1.63]	0.93
Nonsmoker, max-BMI	1.69	[1.62,1.76]	0.95
Smoker, max-BMI	1.87	[1.77,1.98]	0.91

The estimated slope (Coef), its confidence intervals (C.I.), and *R*-squared coefficients for Everyone (Fig. 2), and subgroups.

**Table 2.** Women: Increased incidence associated with prior disease.

Group	Coef	C.I.	*R*-squared
Everyone	1.53	[1.47,1.60]	0.96
Nonsmoker, mid-BMI	1.42	[1.37,1.48]	0.96
Smoker, mid-BMI	1.60	[1.51,1.69]	0.92
Nonsmoker, max-BMI	1.60	[1.52,1.69]	0.94
Smoker, max-BMI	1.74	[1.61,1.87]	0.88

The estimated slope (Coef), its confidence intervals (C.I.), and *R*-squared coefficients for Everyone (Fig. 2), and subgroups.

Subgroups that were expected to include the highest and lowest risk groups were also considered (Tables 1 and 2), these included: nonsmokers in the mid-BMI tertile, smokers in the mid-BMI tertile, nonsmokers in the max-BMI tertile, and smokers in the max-BMI tertile. Statistically significant differences between estimates for men and women (at the 0.05 level), are only found for the nonsmoking, mid-BMI group, that have lower estimates for females. For both men and women, there were statistically significant differences between the group including everyone, and the subgroup of smokers in the max-BMI tertile. For men only, there were statistically significant differences between the group including everyone, and nonsmokers in the max-BMI tertile. Overall, the increase in disease rates above those expected without prior disease tended to be higher for groups that would already be expected to have higher disease risk (e.g. smokers and, or, the top BMI tertile). This above average increase in groups expected to be at higher disease risk was also found in sensitivity studies that did not exclude individuals with reports of pre-existing cardiovascular conditions when they joined the study, finding the estimates were biased upwards, although the estimates’ confidence intervals continued to overlap with those reported in Tables 1 and 2. The sensitivity analyses are reported in online supplementary Tables S3 and S4.

**Fig. 3.**
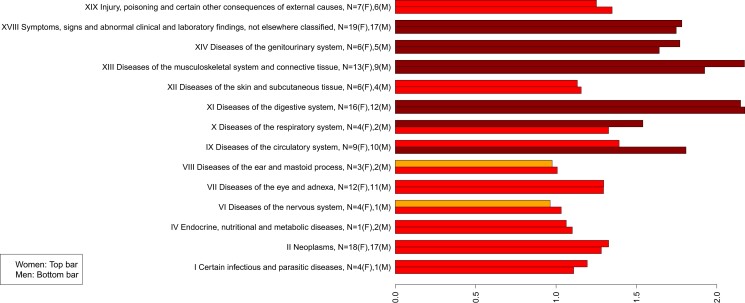
The mean increase in observed cases of disease above that predicted without prior disease, plotted by ICD-10 chapters (women top, men bottom). Numbers of diseases in each bar are indicated by N, with (F) for women and (M) for men. Data for individual diseases are in the online supplementary material.

The ICD-10 coding system groups diseases hierarchically into chapters that are intended to capture the dominant disease types such as cancers (chapter III) or cardiovascular diseases (chapter IX) for example. There were comparatively few diseases representing some ICD-10 chapters (see Fig. 3), but with that caveat, Fig. 3 indicates which disease risks appear to be more susceptible to prior disease. Some chapters such as diseases of the ear (VIII), or of the nervous system (VI), had case numbers similar to that expected without prior disease. In contrast, diseases of the digestive system (XI), circulatory diseases in men (IX), genitourinary diseases (XIV), musculoskeletal diseases (XIII), and unclassified symptoms of potentially unknown origin (XVIII) have case numbers that are all increased in risk by a factor of more than 1.5 above that expected in someone without pre-existing disease.

## Discussion

### Modeling healthcare demand

It had originally been expected that prior disease would increase disease rates, and that these increases would vary substantially between disease types. This could have allowed diseases to be characterized and classified by their sensitivity to prior disease. Instead the increases in disease risk were similar and evenly spread across the diseases considered, and between both men and women. This raises the possibility of using empirical modeling to link estimated disease rates without prior disease, that are considered by conventional epidemiological studies, to those that are observed in practice. Furthermore, in contrast to methods that use present trends to predict future demand (6), the (risk factor-adjusted) models here can be used to explore how changes in population behavior would be expected to modify healthcare demand. This could include the consequences of trends such as increasing BMI, or the expected benefits of policy interventions such as to reduce smoking. Further thought may be needed to determine how this might be done most rigorously through causal counterfactual modeling of potential interventions (24–26), and how best to account for the competing risk of death that is also influenced by exposures and multimorbidity (10).

### Clinical and policy implications

#### Disease risk

The results here emphasize the increased disease risk experienced by individuals with prior or pre-existing disease. These indicate that risk models will need to be modified to account for prior disease, suggesting that risk-stratification and screening may be further improved. This may be particularly true for diseases of the digestive system and musculoskeletal diseases, that were almost twice as prevalent than would be expected based on individuals without prior disease. Several of these diseases were previously identified as being “sporadic” (14), in the sense that statistically at least, they might be avoidable. Follow-up work is needed to determine the causes of these differences between different disease types. For example, are the increases in cardiovascular diseases a simple consequence of e.g. hospital diagnoses for high blood pressure due to attending hospital, or do they represent a more serious deterioration of health. In principle the former concern should have been avoided by considering primary diagnoses that caused hospital admission, as opposed to secondary diagnoses that are made during a hospital stay. Regarding high-risk subgroups, it is well known that otherwise disease-free individuals in subgroups such as smokers with high BMI are at especially high disease risk. Here they are also found to have a higher risk of additional diseases than would be expected based on risks for individuals without prior disease. This is consistent with previous studies (27, 28), and with recent independent studies that have reported associations between increased multimorbidity and smoking in Chinese and Iranian individuals (29–31), and with BMI (32, 33) in studies involving data from Australia, New Zealand, Chile, Finland, Ireland, and England. There is an urgent need to understand if, or how, interventions can lead to long-term health improvements in these high-risk groups. Successful intervention could substantially reduce future healthcare demands, with reduced economic costs and improved individual well-being.

#### Prevention of disease

The reasons for statistically significant differences between the increased risks in subgroups of individuals such as smokers with high BMI, and e.g. nonsmokers with mid-BMI, are presently unknown. It could be that the risk factors are increasing both the underlying disease risk (without prior disease), and the influence of prior disease on your subsequent disease risk. Alternately, the increase in underlying disease risk could lead to a greater number of coexisting disease conditions, that together increase disease risk more than for someone with fewer prior diseases. The particular diseases and mechanisms by which prior disease modifies future disease risk may need to be identified and understood, and more complex modeling of sequences of diseases may be needed. Nonetheless, Fig. 2 suggests that your overall disease risk is driven by your underlying (disease-free) disease risk, and reducing well-understood disease risks will reduce the overall expected burden of disease in old age. Overall, there is an increasingly strong case that policy interventions that can reduce e.g. smoking and BMI, will lead to substantial reductions in healthcare demand.

#### Basic science

There are basic biological questions about how factors such as BMI modify disease risk, and whether these risks can be mitigated by appropriate medication, and whether these observations can lead to new insights about how to treat or prevent disease.

### Limitations

This study considered data in UK Biobank, and approximately 60% of the 400 most common diseases whose incidence rates could be modeled accurately with a Weibull distribution (14). Of these, more stringent fitting requirements detailed in the Methods section reduced the number of diseases considered to 123 in men and 99 in women (222 in total). Future work may be able to improve the modeling of disease onset rates, that would allow a more comprehensive study. The majority of diagnoses relied on hospital records, whose reporting standards and clinical practices are changing with time. This can contribute to year-of-birth (YOB) cohort effects, that could be reduced with sufficient data to allow YOB stratification. A lesser concern involves the small proportion of self-reported cancer and cardiovascular disease diagnoses that could be less accurate than hospital data, and were used to exclude a small number of individuals from the study. Nonetheless, neither of these concerns would be expected to substantially change the trends observed across over 100 diseases, and sensitivity analyses were consistent with this. More general concerns are that the UK Biobank cohort is known to poorly represent the UK population, and both the population and healthcare services will differ from those in other parts of the world. For these reasons, the estimates in Fig. 2 and Tables 1, 2, are likely to be modified for analyses with different cohorts and different collections of diseases. Studies in different cohorts, and with different periods of follow-up, will be needed to explore the generality of these results.

### Future research

#### Methodological improvements

Figure 3 clearly suggests differences in susceptibility of disease risk to prior diseases, based on existing ICD-10 classifications. However, no obvious clusters of diseases were found based on the increased rate of disease, although it is possible that differences could develop if more diseases were able to be included in the study. The figure also suggests that predictions for observed disease rates might be improved by considering predictions within ICD-10 chapters.

#### Generalisability

Follow-up work is needed to explore the generalisability of the model to more diseases, different cohorts, and extrapolation beyond the study period. Provided disease prediction is within the study period, then nonparametric, e.g. proportional hazard models can be used to predict disease risk. Such models would be capable of reliably modeling a wider range of disease types than a Weibull distribution, although they are not suitable for extrapolation to reliably predict disease risk beyond the study period. The UK Biobank cohort is predominately European, comparatively healthy, and has access to a wide range of healthcare services. Follow-up work is needed to determine if, and how, results generalize to different cohorts. When predicting disease risk beyond the end of the study period, extrapolations may need to be combined with a competing-risk model that reliably accounts for risk of death and its modification by risk factors. If this were successful, even for UK cohorts such as UK Biobank, then this would be extremely valuable for allowing the prediction of future healthcare needs. If overall disease risk could be predicted reliably in terms of risk based on age and established risk factors, then it would also allow the consequences of lifestyle changes to be explored and quantified in terms of health and economic cost. This would be valuable for informing both individuals and policy makers, and could lead to stronger public health interventions.

#### Mechanisms and treatments

Independent of the work described here, it is likely that separate approaches will be needed to understand which diseases and what disease mechanisms are responsible for modifying subsequent disease risk. Detailed prediction and understanding of the specific patterns of disease to be experienced by individuals will require more sophisticated approaches, and it is likely that drug use and poly-pharmacy will be important. It is certain that some medications, such as statins, can modify risks of diseases other than those that they are primarily intended to treat.

## Conclusions

### Key innovation

We have identified and demonstrated a statistical method to quantify how much disease risk is increased beyond that expected in healthy individuals without prior disease. This was accomplished by combining a Poisson-Binomial distribution with a Weibull model for the age-related incidence of disease, and applying it to 222 common diseases in the UK Biobank cohort to estimate how much disease risk would be expected after accounting for age and established risk factors.

### Key findings

By comparing these estimates with the total number of new diseases observed, it was found that

1. Age and established risk factors predicted approximately 2/3 of the total new disease cases that were observed.

The disease rates were much greater than would be expected without prior disease, and the results provided a rigorous statistical demonstration of lower disease risk for individuals without any previous disease.

2. The increases in risk were similar for men and women, but ranged between 1 and 2 for different ICD-10 classes of disease types.

Although disease risk differed between ICD-10 classifications of disease types, there were no obvious clusters of diseases in terms of increased risk, that was approximately 1.5 times that expected of individuals without prior disease.

3. The increases in risk were greater for some subgroups such as smokers with high BMI.

### Implications

If confirmed, there are several important implications of overall disease risks being (approximately) proportional to disease risks without prior disease. Firstly, avoiding known risk factors for disease would also reduce the risk, or delay the onset, of multiple diseases in old age. Secondly, it would reaffirm the value of conventional epidemiological studies of disease risk that deliberately avoid potential confounding by prior disease. Thirdly, because the (risk factor-adjusted) model can extrapolate beyond the end of the study period, and the confidence intervals in Fig. 2 are all comparatively narrow, reliable empirical modeling of future disease rates would be possible. These points have not been illustrated quantitatively before, and if confirmed, will be extremely important for predicting healthcare demand and prioritizing healthcare messages.

### Future research

Further studies are needed to determine the generality of the results, both in different cohorts and across a wider range of diseases, and to test the model’s predictive performance beyond the age of the study period. Despite the present limitations, the results provide the first quantitative characterization of how prior disease modifies the incidence rates of a wide range of disease types, and a methodology that can be used or further developed in future studies.

## Materials and methods

### The Poisson-Binomial model

Simple derivations of the key properties of the “Poisson-Binomial” distribution are outlined below, with comprehensive proofs given by others elsewhere (12, 13).

As discussed in the main text, the probability of observing $N_{j}=\sum_{i}N_{ij}$, where $N_{ij}$ is the number of different diseases observed in individual *i*, is given by the “Poisson-Binomial” distribution,


(8) $$\begin{array}{rl} & P\left(\sum_{i}X_{ij}=N_{j}\right)=\sum_{\left\{x_{ij}=0,1\right\}}\delta \left(N_{j}-\sum_{i}x_{ij}\right)\Pi_{i}p_{ij}^{x_{ij}}(1-p_{ij}{)}^{1-x_{ij}} \end{array}$$

where δ(s) is the Dirac delta function that equals 1 when s=0 but is zero otherwise, and the sum is over all $x_{ij}$ for i=1…n. The generating function for 8 is,


(9) $$\begin{array}{rl} G & =\sum_{k=0}^{n}e^{ks}P\left(\sum_{i}X_{ij}=k\right)\\ & =\sum_{k=0}^{n}\sum_{\left\{x_{ij}=0,1\right\}}e^{ks}\delta \left(k=\sum_{i}x_{ij}\right)\Pi_{i}p_{ij}^{x_{ij}}(1-p_{ij}{)}^{1-x_{ij}}\\ & =\sum_{\left\{x_{ij}=0,1\right\}}e^{s\sum_{i}x_{ij}}\Pi_{i}p_{ij}^{x_{ij}}(1-p_{ij}{)}^{1-x_{ij}}\\ & =\sum_{\left\{x_{ij}=0,1\right\}}\Pi_{i}e^{sx_{ij}}p_{ij}^{x_{ij}}(1-p_{ij}{)}^{1-x_{ij}}\\ & =\Pi_{i}(e^{s}p_{ij}+1-p_{ij}) \end{array}$$

where Eq. 8 was included in the second line, the Dirac delta function led to $e^{ks}$ being replaced by $e^{k\sum_{i}x_{ij}}$ when summing over *k* in the third line, the fourth line includes $e^{k\sum_{i}x_{ij}}$ through the product over *i* and *j*, and the final line has summed over each $x_{ij}$ taking values of 0 and 1 with the sum’s evaluation seen most easily by explicitly considering i=1 and factorizing the result. Moments of Eq. 8 can be obtained by taking derivatives of Eq. 9 with respect to *s*, and then evaluating them at s=0, with for example,


(10) $$\begin{array}{rl} E[N_{j}] & ={\frac{\partial G}{\partial s}|}_{s=0}\\ & ={\sum_{i}p_{ij}e^{s}\Pi_{q\neq i}(e^{s}p_{qj}+1-p_{qj})|}_{s=0}\\ & =\sum_{ij}p_{ij}\Pi_{q\neq i}(p_{qj}+1-p_{qj})\\ & =\sum_{i}p_{ij} \end{array}$$

Similarly, the second moment is


(11) $$\begin{array}{rl} E[N_{j}^{2}] & ={\frac{\partial^{2}G}{\partial s^{2}}|}_{s=0}\\ & ={\frac{\partial }{\partial s}\sum_{i}p_{ij}e^{s}\Pi_{q\neq i}(e^{s}p_{qj}+1-p_{qj})|}_{s=0}\\ & ={\frac{\partial }{\partial s}\sum_{i}\frac{\;p_{ij}e^{s}}{e^{s}p_{ij}+1-p_{ij}}\Pi_{q}(e^{s}p_{qj}+1-p_{qj})|}_{s=0}\\ & =-\sum_{i}p_{ij}^{2}+\sum_{i}p_{ij}+{\left(\sum_{i}p_{ij}\right)}^{2} \end{array}$$

where in the final line the first term arises from the derivative of the denominator of the first term in line 3, the second term from the derivative of the numerator of the first term in line 3, and the final term is from the derivative of the product in line 3 similarly to evaluating $E[N_{j}]$. Combining Eq. 10 with Eq. 11, the variance can be evaluated as


(12) $$E[N_{j}^{2}]-E[N_{j}{]}^{2}=\sum_{i}p_{ij}(1-p_{ij})$$

$E[N_{j}]$ and $\text{Var}[N_{j}]$ can alternatively be written as $E[N_{j}]=n\bar{\;p}$ and $Var[N_{j}]=n\bar{\text{Var}(p)}$, where the bars denote averages. If $p_{ij}\ll 1$, then the variance Eq. 12 has $\text{Var}[N_{j}]≃\sum_{i}p_{ij}=E[N_{j}]$, as would be the case for a Poisson distribution with rate $\lambda_{j}=\sum_{i}p_{ij}$ and $P(N_{j})=\lambda_{j}^{N_{j}}e^{-\lambda_{j}}/N_{j}!$. In fact, when $p_{ij}\ll 1$, the Poisson-Binomial distribution tends to the Poisson distribution (12), as can be seen from the moment generation function Eq. 9, that approximates the Possion distribution for $p_{ij}\to 0$. Using Eq. 9 and expanding in terms of $p_{ij}$,


(13) $$\begin{array}{rl} G & =\Pi_{i}(1+p_{ij}(e^{s}-1))\\ & =\exp\;\left\{\sum_{i}\log\;(1+p_{ij}(e^{s}-1))\right\}\\ & =\exp\;\left\{\sum_{i}p_{ij}(e^{s}-1)+O(p_{ij}^{2})\right\}\\ & =\exp\;((e^{s}-1)\lambda_{j}+O(p_{ij}^{2})) \end{array}$$

where $\lambda_{j}=\sum_{i}p_{ij}$, and the generating function for a Poisson distribution is $\exp\;((e^{s}-1)\lambda_{j})$. The above argument suggests that provided $p_{ij}\ll 1$, then a Poisson distribution can model the number of events due to *n* independent processes that each have different but low probabilities $p_{ij}$ of occurring.

## Supplementary Material

pgad279_Supplementary_Data

## Data Availability

UK Biobank data can be accessed by application through www.ukbiobank.ac.uk. UK Biobank has approval by the Research Ethics Committee under approval number 16/NW/0274. UK Biobank obtained participant’s consent for the data to be used for health-related research, and all methods were performed in accordance with the relevant guidelines and regulations. R code used to produce figures from summary data is available from https://osf.io/mahqb/. R packages used in this study include survival (34), grr (35), data.table (36), and maxLik (37).

## Preprints

This manuscript was posted as a preprint on medRxiv: https://doi.org/10.1101/2023.01.20.23284838.
