# Supplementary material for: How much disease risk is due to old age and established risk factors?
Source: PNAS Nexus. 2023 Sep 12;2(9):pgad279. doi: 10.1093/pnasnexus/pgad279 (PMC10496869; doi:10.1093/pnasnexus/pgad279)
Supplement: pgad279_Supplementary_Data [file pgad279_supplementary_data.pdf]

Supplementary material for: How much multi-morbidity is due to old age and established risk factors?

Anthony J. Webster

Nuffield Department of Population Health, Lindgren Group, Big Data Institute, Old Road Campus, University of Oxford, Oxford, OX3 7LF, UK.

This supplementary material lists the diseases studied in the main article, and the estimates associated with them. Diseases studied in men and women are listed separately, with diseases studied in women listed first. It also provides details of sensitivity analyses referred to in the main text.

Table S1: Women - predicted and observed numbers of cases

ICD	Disease	Predicted	Obs (FIC)	Obs (all)	sd	Z (FIC)	Obs(all)/Pred
A08(F)	A08 Viral and other specified intestinal infections	369	377	441	19.2	-0.403	1.194
C15(F)	C15 Malignant neoplasm of oesophagus	162	151	205	12.7	0.854	1.267
C18(F)	C18 Malignant neoplasm of colon	926	908	1313	30.4	0.577	1.419
C20(F)	C20 Malignant neoplasm of rectum	319	318	463	17.9	0.06	1.451
C25(F)	C25 Malignant neoplasm of pancreas	275	256	383	16.6	1.142	1.393
C43(F)	C43 Malignant melanoma of skin	699	669	922	26.4	1.146	1.318
C50(F)	C50 Malignant neoplasm of breast	5539	5740	6638	74.4	-2.704	1.198
C56(F)	C56 Malignant neoplasm of ovary	494	476	713	22.2	0.803	1.444
C64(F)	C64 Malignant neoplasm of kidney, except renal pelvis	253	234	316	15.9	1.19	1.249
C67(F)	C67 Malignant neoplasm of bladder	335	312	419	18.3	1.244	1.252
C71(F)	C71 Malignant neoplasm of brain	176	167	255	13.3	0.647	1.452
C833(F)	C83.3 Large cell (diffuse)	158	147	261	12.6	0.837	1.657
C90(F)	C90 Multiple myeloma and malignant plasma cell neoplasms	233	221	187	15.3	0.769	0.804
D03(F)	D03 Melanoma in situ	271	260	371	16.5	0.657	1.37
D04(F)	D04 Carcinoma in situ of skin	197	182	304	14	1.086	1.541
D05(F)	D05 Carcinoma in situ of breast	1001	981	1255	31.6	0.619	1.254
D12(F)	D12 Benign neoplasm of colon, rectum, anus and anal canal	4963	4783	5773	70.4	2.554	1.163
D351(F)	D35.1 Parathyroid gland	230	221	305	15.2	0.582	1.327
E21(F)	E21 Hyperparathyroidism and other disorders of parathyroid gland	316	303	335	17.8	0.754	1.059
E871(F)	E87.1 Hypo-osmolality and hyponatraemia	329	341	351	18.1	-0.689	1.068
G45(F)	G45 Transient cerebral ischaemic attacks and related syndromes	869	773	837	29.5	3.255	0.963
H020(F)	H02.0 Entropion and trichiasis of eyelid	219	206	263	14.8	0.908	1.198
H024(F)	H02.4 Ptosis of eyelid	418	406	543	20.4	0.577	1.3
H042(F)	H04.2 Epiphora	237	236	330	15.4	0.053	1.393
H045(F)	H04.5 Stenosis and insufficiency of lachrymal passages	435	423	520	20.9	0.563	1.196
H250(F)	H25.0 Senile incipient cataract	1123	1113	1506	33.5	0.3	1.341
H259(F)	H25.9 Senile cataract, unspecified	650	640	901	25.5	0.406	1.385
H268(F)	H26.8 Other specified cataract	489	504	702	22.1	-0.658	1.434
H33(F)	H33 Retinal detachments and breaks	765	780	979	27.7	-0.541	1.28
H34(F)	H34 Retinal vascular occlusions	121	115	147	11	0.531	1.217
H353(F)	H35.3 Degeneration of macula and posterior pole	804	796	1041	28.4	0.277	1.295
H40(F)	H40 Glaucoma	926	928	1131	30.4	-0.081	1.222
H610(F)	H61.0 Perichondritis of external ear	131	125	129	11.5	0.546	0.983
H811(F)	H81.1 Benign paroxysmal vertigo	183	171	177	13.5	0.873	0.968
I10(F)	I10 Essential (primary) hypertension	505	562	639	22.5	-2.539	1.265
I20(F)	I20 Angina pectoris	1076	1118	1533	32.8	-1.268	1.424
I251(F)	I25.1 Atherosclerotic heart disease	1662	1773	2576	40.8	-2.723	1.55
I259(F)	I25.9 Chronic ischaemic heart disease, unspecified	232	249	329	15.2	-1.139	1.42
I35(F)	I35 Nonrheumatic aortic valve disorders	267	283	380	16.3	-0.991	1.424
I44(F)	I44 Atrioventricular and left bundle-branch block	275	301	392	16.6	-1.544	1.424
I48(F)	I48 Atrial fibrillation and flutter	1925	2099	2327	43.9	-3.956	1.209
I61(F)	I61 Intracerebral haemorrhage	198	219	260	14.1	-1.513	1.315
I64(F)	I64 Stroke, not specified as haemorrhage or infarction	132	129	180	11.5	0.272	1.362
I95(F)	I95 Hypotension	267	305	409	16.4	-2.301	1.53
J22(F)	J22 Unspecified acute lower respiratory infection	1398	1513	1920	37.4	-3.081	1.374
J47(F)	J47 Bronchiectasis	203	190	346	14.2	0.902	1.706
K20(F)	K20 Oesophagitis	953	958	1881	30.9	-0.163	1.974
K21(F)	K21 Gastro-oesophageal reflux disease	2638	2808	5158	51.4	-3.301	1.955

ICD	Disease	Predicted	Obs (FIC)	Obs (all)	sd	Z (FIC)	Obs(all)/Pred
K222(F)	K22.2 Oesophageal obstruction	176	194	436	13.3	-1.391	2.483
K227(F)	K22.7 Barrett's oesophagus	316	331	1086	17.8	-0.864	3.44
K25(F)	K25 Gastric ulcer	461	447	950	21.5	0.659	2.06
K29(F)	K29 Gastritis and duodenitis	3888	4028	7466	62.4	-2.252	1.92
K317(F)	K31.7 Polyp of stomach and duodenum	800	826	1962	28.3	-0.928	2.453
K40(F)	K40 Inguinal hernia	446	438	765	21.1	0.391	1.714
K44(F)	K44 Diaphragmatic hernia	2163	2191	4500	46.5	-0.601	2.08
K57(F)	K57 Diverticular disease of intestine	5007	5098	9004	70.8	-1.284	1.798
K621(F)	K62.1 Rectal polyp	917	942	1828	30.3	-0.834	1.994
K635(F)	K63.5 Polyp of colon	2678	2772	5094	51.8	-1.814	1.902
L03(F)	L03 Cellulitis	1518	1624	1739	39	-2.734	1.146
L57(F)	L57 Skin changes due to chronic exposure to nonionising radiation	908	897	1011	30.1	0.37	1.113
L82(F)	L82 Seborrhoeic keratosis	907	909	1020	30.1	-0.069	1.125
L85(F)	L85 Other epidermal thickening	219	219	251	14.8	0.007	1.146
M15(F)	M15 Polyarthrosis	584	567	1675	24.2	0.688	2.87
M16(F)	M16 Coxarthrosis [arthrosis of hip]	4386	4410	6502	66.2	-0.368	1.483
M17(F)	M17 Gonarthrosis [arthrosis of knee]	3666	3695	7091	60.5	-0.487	1.935
M19(F)	M19 Other arthrosis	1313	1292	2939	36.2	0.579	2.238
M204(F)	M20.4 Other hammer toe(s) (acquired)	268	252	578	16.4	0.976	2.157
M43(F)	M43 Other deforming dorsopathies	195	200	565	14	-0.328	2.891
M48(F)	M48 Other spondylopathies	539	552	1481	23.2	-0.549	2.746
M720(F)	M72.0 Palmar fascial fibromatosis [Dupuytren]	540	544	696	23.2	-0.156	1.288
M751(F)	M75.1 Rotator cuff syndrome	713	715	1384	26.7	-0.07	1.941
N13(F)	N13 Obstructive and reflux uropathy	277	330	621	16.6	-3.175	2.241
N17(F)	N17 Acute renal failure	361	399	664	19	-1.994	1.839
N35(F)	N35 Urethral stricture	380	377	643	19.5	0.141	1.693
N390(F)	N39.0 Urinary tract infection, site not specified	1781	1879	2964	42.2	-2.313	1.664
N81(F)	N81 Female genital prolapse	3746	3781	5295	61.2	-0.567	1.413
R040(F)	R04.0 Epistaxis	345	330	513	18.6	0.825	1.486
R060(F)	R06.0 Dyspnoea	750	806	1521	27.4	-2.03	2.027
R072(F)	R07.2 Precordial pain	1055	1079	2086	32.5	-0.724	1.976
R074(F)	R07.4 Chest pain, unspecified	3382	3512	5650	58.2	-2.229	1.67
R13(F)	R13 Dysphagia	1344	1415	2462	36.7	-1.935	1.832
R15(F)	R15 Faecal incontinence	437	432	732	20.9	0.261	1.673
R190(F)	R19.0 Intra-abdominal and pelvic swelling, mass and lump	158	172	279	12.6	-1.076	1.761
R194(F)	R19.4 Change in bowel habit	3135	3277	4844	56	-2.539	1.545
R195(F)	R19.5 Other fecal abnormalities	509	508	754	22.6	0.064	1.48
R410(F)	R41.0 Disorientation, unspecified	183	202	380	13.5	-1.39	2.074
R42(F)	R42 Dizziness and giddiness	501	480	912	22.4	0.954	1.819
R55(F)	R55 Syncope and collapse	1424	1435	2332	37.7	-0.283	1.637
R634(F)	R63.4 Abnormal weight loss	454	451	824	21.3	0.134	1.816
R79(F)	R79 Other abnormal findings of blood chemistry	159	152	279	12.6	0.561	1.754
R91(F)	R91 Abnormal findings on diagnostic imaging of lung	337	374	659	18.4	-2.029	1.957
R933(F)	R93.3 Abnormal findings on diag. imag. of parts of digest. tract	266	293	590	16.3	-1.673	2.22
R945(F)	R94.5 Abnormal results of liver function studies	171	168	268	13.1	0.191	1.572
S010(F)	S01.0 Open wound of scalp	138	140	181	11.8	-0.141	1.308
S32(F)	S32 Fracture of lumbar spine and pelvis	492	485	660	22.2	0.314	1.342
S42(F)	S42 Fracture of shoulder and upper arm	907	895	1133	30.1	0.395	1.249
S52(F)	S52 Fracture of forearm	3216	3280	3709	56.7	-1.124	1.153
S72(F)	S72 Fracture of femur	1363	1385	1732	36.9	-0.603	1.271
S82(F)	S82 Fracture of lower leg, including ankle	1982	2013	2347	44.5	-0.698	1.184

Table S2: Men - predicted and observed numbers of cases

ICD	Disease	Predicted	Obs (FIC)	Obs (all)	sd	Z (FIC)	Obs(all)/Pred
A08(M)	A08 Viral and other specified intestinal infections	271	249	284	16.5	1.333	1.048
A09(M)	A09 Diarrhoea and gastro-enteritis of presumed infectious origin	1948	2067	2207	44.1	-2.705	1.133
B07(M)	B07 Viral warts	188	197	206	13.7	-0.648	1.095
B37(M)	B37 Candidiasis	174	181	202	13.2	-0.549	1.162
C15(M)	C15 Malignant neoplasm of oesophagus	456	417	554	21.4	1.831	1.215
C16(M)	C16 Malignant neoplasm of stomach	246	230	434	15.7	1.026	1.764
C18(M)	C18 Malignant neoplasm of colon	1119	1055	1459	33.5	1.923	1.303
C20(M)	C20 Malignant neoplasm of rectum	557	534	778	23.6	0.973	1.397
C22(M)	C22 Malignant neoplasm of liver and intrahepatic bile ducts	196	163	240	14	2.357	1.225
C25(M)	C25 Malignant neoplasm of pancreas	306	279	424	17.5	1.542	1.386
C43(M)	C43 Malignant melanoma of skin	667	634	839	25.8	1.27	1.258
C45(M)	C45 Mesothelioma	203	187	217	14.3	1.15	1.067
C64(M)	C64 Malignant neoplasm of kidney, except renal pelvis	445	420	576	21.1	1.189	1.294
C71(M)	C71 Malignant neoplasm of brain	271	261	330	16.4	0.582	1.22
C833(M)	C83.3 Large cell (diffuse)	182	167	319	13.5	1.127	1.751
C90(M)	C90 Multiple myeloma and malignant plasma cell neoplasms	289	258	226	17	1.83	0.782
C911(M)	C91.1 Chronic lymphocytic leukaemia	144	107	137	12	3.073	0.952
D03(M)	D03 Melanoma in situ	261	235	362	16.1	1.584	1.389
D04(M)	D04 Carcinoma in situ of skin	195	160	296	14	2.482	1.521
D23(M)	D23 Other benign neoplasms of skin	603	554	707	24.6	2.003	1.172
D414(M)	D41.4 Bladder	215	187	258	14.7	1.912	1.2
D46(M)	D46 Myelodysplastic syndromes	123	115	144	11.1	0.718	1.171
D50(M)	D50 Iron deficiency anaemia	2199	2103	2297	46.9	2.043	1.045
E871(M)	E87.1 Hypo-osmolality and hyponatraemia	224	229	247	15	-0.315	1.101
G20(M)	G20 Parkinson’s disease	261	255	258	16.1	0.359	0.989
G45(M)	G45 Transient cerebral ischaemic attacks and related syndromes	1026	907	966	32	3.729	0.941
G560(M)	G56.0 Carpal tunnel syndrome	1801	1760	1820	42.4	0.958	1.011
G62(M)	G62 Other polyneuropathies	153	159	182	12.4	-0.459	1.187
H020(M)	H02.0 Entropion and trichiasis of eyelid	316	296	362	17.8	1.124	1.146
H021(M)	H02.1 Ectropion of eyelid	211	204	277	14.5	0.493	1.312
H023(M)	H02.3 Blepharochalasis	127	112	148	11.3	1.296	1.169
H024(M)	H02.4 Ptosis of eyelid	239	225	318	15.5	0.903	1.331
H042(M)	H04.2 Epiphora	114	111	157	10.7	0.266	1.379
H045(M)	H04.5 Stenosis and insufficiency of lachrymal passages	172	153	226	13.1	1.417	1.317
H250(M)	H25.0 Senile incipient cataract	742	689	970	27.2	1.949	1.307
H258(M)	H25.8 Other senile cataract	1045	991	1372	32.3	1.676	1.313
H268(M)	H26.8 Other specified cataract	306	305	436	17.5	0.031	1.427
H34(M)	H34 Retinal vascular occlusions	138	136	178	11.8	0.192	1.287
H353(M)	H35.3 Degeneration of macula and posterior pole	465	441	604	21.6	1.099	1.3
H40(M)	H40 Glaucoma	734	703	922	27.1	1.151	1.256
H610(M)	H61.0 Perichondritis of external ear	135	123	126	11.6	1.02	0.934
H65(M)	H65 Nonsuppurative otitis media	214	207	246	14.6	0.503	1.148
H830(M)	H83.0 Labyrinthitis	203	182	191	14.2	1.474	0.941
I20(M)	I20 Angina pectoris	1208	1329	2069	34.8	-3.486	1.713
I24(M)	I24 Other acute ischaemic heart diseases	206	234	492	14.3	-1.96	2.39
I259(M)	I25.9 Chronic ischaemic heart disease, unspecified	251	277	415	15.9	-1.623	1.652
I34(M)	I34 Nonrheumatic mitral valve disorders	188	208	332	13.7	-1.488	1.769
I35(M)	I35 Nonrheumatic aortic valve disorders	350	399	662	18.7	-2.607	1.89
I50(M)	I50 Heart failure	385	434	885	19.6	-2.495	2.298
I61(M)	I61 Intracerebral haemorrhage	249	285	343	15.8	-2.286	1.378
I62(M)	I62 Other nontraumatic intracranial haemorrhage	138	174	218	11.8	-3.04	1.577
I64(M)	I64 Stroke, not specified as haemorrhage or infarction	179	196	288	13.4	-1.256	1.607
J22(M)	J22 Unspecified acute lower respiratory infection	1343	1440	1881	36.6	-2.649	1.401
J33(M)	J33 Nasal polyp	667	682	770	25.8	-0.591	1.155
J84(M)	J84 Other interstitial pulmonary diseases	236	231	324	15.4	0.351	1.371
J90(M)	J90 Pleural effusion, not elsewhere classified	525	552	724	22.9	-1.197	1.38

ICD	Disease	Predicted	Obs (FIC)	Obs (all)	sd	Z (FIC)	Obs(all)/Pred
K20(M)	K20 Oesophagitis	871	879	1691	29.5	-0.277	1.942
K21(M)	K21 Gastro-oesophageal reflux disease	1857	1935	3629	43.1	-1.818	1.955
K221(M)	K22.1 Ulcer of oesophagus	449	430	887	21.2	0.913	1.974
K227(M)	K22.7 Barrett's oesophagus	544	565	1788	23.3	-0.879	3.284
K25(M)	K25 Gastric ulcer	323	298	701	18	1.409	2.168
K26(M)	K26 Duodenal ulcer	254	239	510	15.9	0.938	2.008
K29(M)	K29 Gastritis and duodenitis	2577	2494	4800	50.8	1.636	1.863
K317(M)	K31.7 Polyp of stomach and duodenum	317	305	928	17.8	0.673	2.928
K40(M)	K40 Inguinal hernia	6179	6436	8980	78.6	-3.265	1.453
K44(M)	K44 Diaphragmatic hernia	1140	1155	2510	33.8	-0.443	2.202
K590(M)	K59.0 Constipation	638	698	1446	25.3	-2.393	2.268
K621(M)	K62.1 Rectal polyp	1075	1121	2134	32.8	-1.4	1.985
K80(M)	K80 Cholelithiasis	1223	1200	2645	35	0.659	2.163
K81(M)	K81 Cholecystitis	277	268	696	16.7	0.562	2.509
K921(M)	K92.1 Melaena	284	285	616	16.8	-0.081	2.172
K922(M)	K92.2 Gastro-intestinal haemorrhage, unspecified	1270	1258	2421	35.6	0.326	1.907
L03(M)	L03 Cellulitis	1975	1898	2053	44.4	1.742	1.039
L57(M)	L57 Skin changes due to chronic exposure to nonionising radiation	815	756	902	28.6	2.073	1.107
L81(M)	L81 Other disorders of pigmentation	174	175	203	13.2	-0.04	1.164
L82(M)	L82 Seborrhoeic keratosis	820	811	927	28.6	0.325	1.13
L85(M)	L85 Other epidermal thickening	211	202	238	14.5	0.609	1.129
L905(M)	L90.5 Scar conditions and fibrosis of skin	280	313	383	16.7	-1.991	1.369
M10(M)	M10 Gout	195	188	307	13.9	0.468	1.578
M13(M)	M13 Other arthritis	203	208	483	14.3	-0.319	2.374
M15(M)	M15 Polyarthrosis	331	324	865	18.2	0.384	2.613
M16(M)	M16 Coxarthrosis [arthrosis of hip]	2934	2899	4132	54.2	0.652	1.408
M17(M)	M17 Gonarthrosis [arthrosis of knee]	3139	3036	5461	56	1.835	1.74
M18(M)	M18 Arthrosis of first carpometacarpal joint	113	117	284	10.6	-0.353	2.508
M19(M)	M19 Other arthrosis	766	727	1527	27.7	1.406	1.994
M47(M)	M47 Spondylosis	328	298	854	18.1	1.675	2.601
M48(M)	M48 Other spondylopathies	533	507	1138	23.1	1.118	2.136
M653(M)	M65.3 Trigger finger	451	426	678	21.2	1.164	1.504
M720(M)	M72.0 Palmar fascial fibromatosis [Dupuytren]	1636	1625	2003	40.4	0.27	1.224
M751(M)	M75.1 Rotator cuff syndrome	862	871	1473	29.4	-0.298	1.708
M796(M)	M79.6 Pain in limb	752	714	1222	27.4	1.371	1.626
N13(M)	N13 Obstructive and reflux uropathy	593	682	1078	24.4	-3.658	1.818
N17(M)	N17 Acute renal failure	659	658	953	25.7	0.047	1.446
N21(M)	N21 Calculus of lower urinary tract	193	207	419	13.9	-1.02	2.173
N320(M)	N32.0 Bladder-neck obstruction	359	364	656	19	-0.256	1.827
N41(M)	N41 Inflammatory diseases of prostate	537	553	766	23.2	-0.676	1.426
N43(M)	N43 Hydrocele and spermatocele	603	590	700	24.6	0.531	1.161
R001(M)	R00.1 Bradycardia, unspecified	219	202	346	14.8	1.175	1.577
R040(M)	R04.0 Epistaxis	469	409	635	21.7	2.791	1.353
R060(M)	R06.0 Dyspnoea	651	647	1168	25.5	0.142	1.795
R101(M)	R10.1 Pain localised to upper abdomen	1155	1183	2005	34	-0.829	1.736
R13(M)	R13 Dysphagia	907	932	1529	30.1	-0.828	1.686
R194(M)	R19.4 Change in bowel habit	1664	1711	2559	40.8	-1.145	1.538
R195(M)	R19.5 Other fecal abnormalities	329	335	547	18.1	-0.324	1.662
R296(M)	R29.6 Tendency to fall, not elsewhere classified	181	178	437	13.4	0.195	2.419
R31(M)	R31 Unspecified haematuria	2492	2558	3659	49.9	-1.331	1.469
R33(M)	R33 Retention of urine	1205	1333	2145	34.7	-3.694	1.78
R35(M)	R35 Polyuria	295	288	488	17.2	0.379	1.657
R39(M)	R39 Other symptoms and signs involving the urinary system	619	639	1082	24.9	-0.798	1.748
R410(M)	R41.0 Disorientation, unspecified	215	248	489	14.7	-2.223	2.27
R42(M)	R42 Dizziness and giddiness	491	474	868	22.2	0.758	1.769
R55(M)	R55 Syncope and collapse	1732	1654	2628	41.6	1.874	1.517
R568(M)	R56.8 Other and unspecified convulsions	299	320	550	17.3	-1.193	1.837
R634(M)	R63.4 Abnormal weight loss	328	320	563	18.1	0.466	1.714
R69(M)	R69 Unknown and unspecified causes of morbidity	969	1060	1525	31.1	-2.933	1.574
R933(M)	R93.3 Abnormal findings on diag. imag. of parts of digest. tract	204	230	429	14.3	-1.792	2.099

ICD	Disease	Predicted	Obs (FIC)	Obs (all)	sd	Z (FIC)	Obs(all)/Pred
S008(M)	S00.8 Superficial injury of other parts of head	151	147	231	12.3	0.364	1.525
S010(M)	S01.0 Open wound of scalp	163	151	240	12.8	0.971	1.469
S018(M)	S01.8 Open wound of other parts of head	240	237	356	15.5	0.178	1.485
S065(M)	S06.5 Traumatic subdural haemorrhage	187	175	244	13.7	0.885	1.304
S32(M)	S32 Fracture of lumbar spine and pelvis	306	281	394	17.5	1.438	1.287
S72(M)	S72 Fracture of femur	667	651	863	25.8	0.612	1.294
S761(M)	S76.1 Injury of quadriceps muscle and tendon	206	195	222	14.4	0.785	1.076

Sensitivity analyses

Participants were originally excluded from the study if they had a self-report of heart attack, heart failure, a heart or cardiac problem, stroke, arterial or pulmonary embolism, or of subarachnoid haemorrhage. They were also excluded if they had a primary hospital diagnosis of a cardiovascular problem for which they were admitted for treatment. A primary hospital diagnosis is the reason why a patient would be admitted to hospital and is likely to lead to treatment, and either the disease or its treatment would be expected to influence an individual's health, confounding the subsequent results. These restrictions were comparatively strict, so the analyses were repeated for data with exclusions for prior cancer, but without exclusions for a previous report of cardiovascular disease. The confidence intervals overlap with those of the main text, but the estimates below are all as big or greater. This is consistent with the hypothesis that pre-existing cardiovascular disease (CVD) would increase your risk of subsequent diseases, and supports the decision to exclude participants with pre-existing CVD from the study. However, although this leads to a better scientific study with reduced risk of confounding by pre-existing disease, it does risk studying a healthier population than is typical, and this may be imperfectly adjusted for.

Men: Increased incidence associated with prior disease

Group	Coef	C.I.	R-squared
Everyone	1.6	[1.54,1.66]	0.96
Non-smoker, mid-BMI	1.52	[1.47,1.57]	0.97
Smoker, mid-BMI	1.6	[1.52,1.69]	0.92
Non-smoker, max-BMI	1.78	[1.7,1.87]	0.94
Smoker, max-BMI	1.96	[1.84,2.08]	0.9

Table S3: The estimated increase in observed disease cases above those expected without prior disease (Coef), its confidence intervals (C.I.), and R-squared coefficients for the fits. Data shown for Everyone and important sub-groups.

Women: Increased incidence associated with prior disease

Group	Coef	C.I.	R-squared
Everyone	1.55	[1.48,1.63]	0.95
Non-smoker, mid-BMI	1.48	[1.42,1.54]	0.96
Smoker, mid-BMI	1.69	[1.58,1.8]	0.91
Non-smoker, max-BMI	1.69	[1.6,1.79]	0.93
Smoker, max-BMI	1.94	[1.8,2.08]	0.89

Table S4: The estimated increase in observed disease cases above those expected without prior disease (Coef), its confidence intervals (C.I.), and R-squared coefficients for the fits. Data shown for Everyone and important sub-groups.

Code and data availability

UK Biobank data can be accessed by application through www.ukbiobank.ac.uk. UK Biobank has approval by the Research Ethics Committee (REC) under approval number 16/NW/0274. UK Biobank obtained participant's consent for the data to be used for health-related research, and all methods were performed in accordance with the relevant guidelines and regulations. R code used to produce figures from summary data will be made available from:

`https://osf.io/mahqb/`

R packages used in this study include survival[1], grr[2], data.table[3], and maxLik[4].

Acknowledgments

This manuscript was posted as a preprint on medRxiv: https://doi.org/10.1101/2023.01.20.23284838. This research has been conducted using data from UK Biobank, a major biomedical database, under application number 42583. This research was supported by an intermediate research fellowship from the Nuffield Department of Population Health (NDPH), University of Oxford.

References

[1] Terry M Therneau. *A Package for Survival Analysis in R*, 2023. R package version 3.5-0.

[2] Craig Varrichio. *grr: Alternative Implementations of Base R Functions*, 2016. R package version 0.9.5.

[3] Matt Dowle and Arun Srinivasan. *data.table: Extension of 'data.frame'*, 2021. R package version 1.14.0.

[4] Arne Henningsen and Ott Toomet. maxlik: A package for maximum likelihood estimation in R. *Computational Statistics*, 26(3):443–458, 2011.
